# Triple Teeth: Report of an Unusual Case

**DOI:** 10.1155/2012/735925

**Published:** 2012-12-17

**Authors:** Prashant Babaji, M. A. Prasanth, Ajith R. Gowda, Soumya Ajith, Henston D'Souza, K. P. Ashok

**Affiliations:** ^1^Department of Pedodontics, SPPIDMS Dental College, Utter Pradesh, Lucknow 226001, India; ^2^Department of Pedodontics, Vyas Dental College & Hospital, Jodhpur 342001, India; ^3^Department of Orthodontics, Sri Hasanamba Dental College, Hassan 573201, India; ^4^Department of Periodontics, Sri Hasanamba Dental College, Hassan 573201, India; ^5^Department of Conservative Dentistry, Vyas Dental College, Jodhpur 342001, India; ^6^Department of Periodontics, Vyas Dental College, Jodhpur 342001, India

## Abstract

Fusion or synodontia is a union of two or more than two developing teeth. Commonly fusion occurs between teeth of the same dentition, mixed dentition, or between normal and supernumerary teeth. Fused primary teeth present with several clinical problems like caries, periodontal problem, arch asymmetry, delayed eruption, ectopic eruption of succedaneous teeth, aesthetic, and other complications. This paper presents a rare and unusual case of triple teeth in mandibular primary dentition.

## 1. Introduction

 The word synodontia or fusion means union of two or more teeth [1, 2]. It is also known as double teeth, double formations, conjoined teeth, joined teeth, fused teeth, or dental twinning [3]. There is no gender predilection [2]. The prevalence of fused teeth in primary dentition is 0.5% to 1% compared to 0.01% to 0.2% in permanent dentition [4, 5]. Fused teeth have predilection for mandible over maxilla, unilateral (0.05%) over bilateral (0.02%), and deciduous (0.5%) over permanent dentition (0.1%) [4, 5]. Fusion is common in anterior teeth that to incisor and canine region but isolated cases involving molars or its association with Russel-Silver syndrome are also reported [1, 2]. 

## 2. Case Report

**Figure 1:**
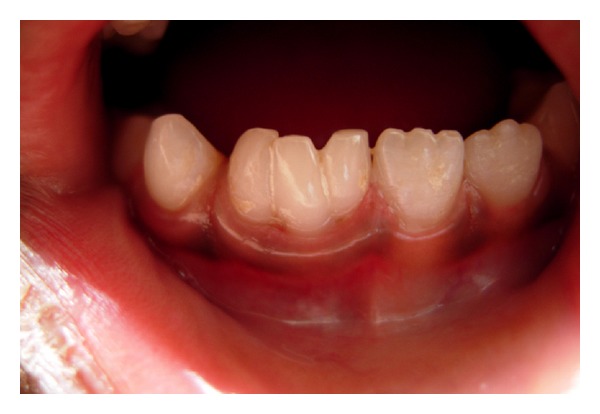
Image of lower arch showing fusion of two primary incisors with supernumerary tooth.

**Figure 2:**
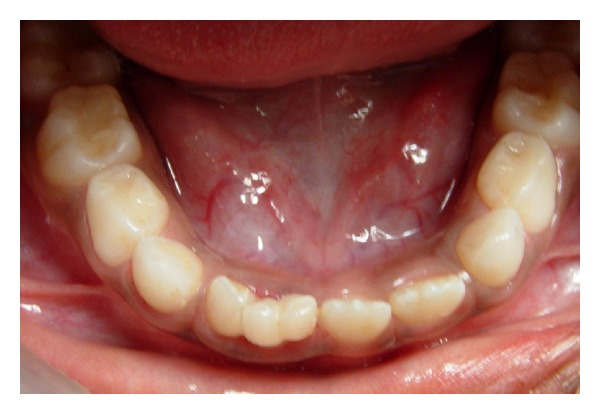
Image of lower arch showing triple teeth on right side and permanent central and lateral incisors on left side.

**Figure 3:**
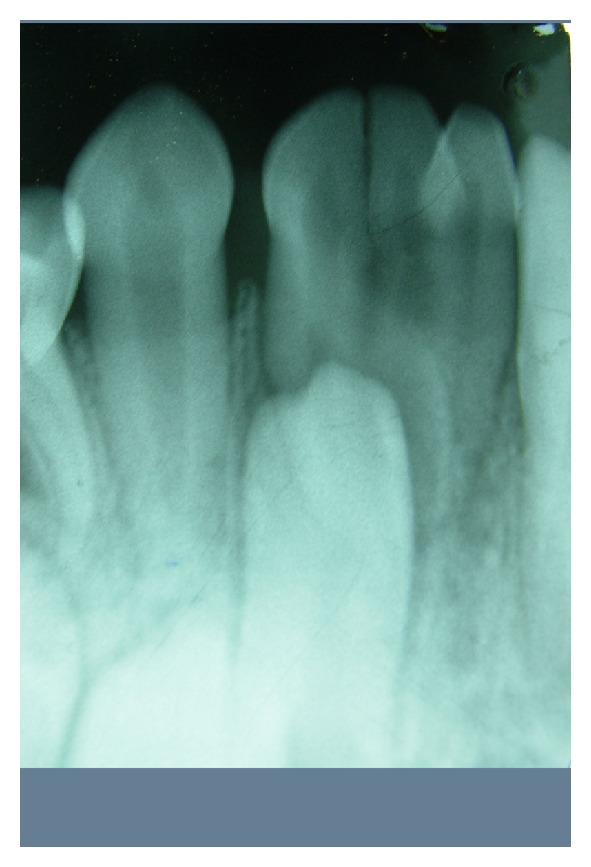
Intraoral periapical radiograph showing fusion of two primary incisors with supernumerary tooth, with separate pulp chamber and root canals, erupting succedaneous lateral incisor, canine, and absence of central incisor on right side.

 A six-year-old boy was reported with chief complaint of large tooth in the lower jaw. His medical and family history was noncontributory. Intraoral examination revealed unusual presence of large fused triple teeth at incisor region on right side and erupted permanent central and lateral incisors on left side of mandible (Figures 1 and 2). There was deep vertical groove at the union without caries or any other dental abnormalities. Intraoral periapical radiograph shows fusion of two primary incisors with supernumerary tooth (triple teeth), with separate pulp chamber and root canals, erupting succedaneous lateral incisor and canine but absence of central incisor on right side as its position and mesiodistal dimension resemble erupted lateral incisor on left side (Figure 3). It was diagnosed as an unusual case of triple teeth in deciduous anterior region which is of incomplete fusion. Since the fused teeth were asymptomatic, recall examination was planned until exfoliation of triple teeth.

## 3. Discussion

 Fusion of two distinct developing teeth can occur at any stage of tooth development. It can be seen with complete or incomplete fusion. If this contact occurs early at least before calcification begins, the two teeth may be completely united to form single tooth. If the contact of teeth occurs later, when a portion of the tooth crown has completed its formation, there may be union of the roots only. There will be different clinical and radiographic appearance depending on the stage of teeth development during fusion [2]. Fused teeth are joined by the dentine; pulp chambers and canals may be linked or separated depending on the developmental stage when union occurs [6]. Commonly fusion occurs between teeth of the same dentition, mixed dentition, or between normal and supernumerary teeth [1, 5, 6]. Supernumerary tooth is less commonly seen in the primary dentition with prevalence rate of 0.2–3.8% and is usually of supplemental type [1, 7]. Supernumerary tooth develops as a consequence of the proliferation of epithelial cells from dental lamina [1]. The worldwide incidence of fused teeth ranges from 0.14% to 5.0% [4]. Asian and Asian-derived populations showed higher prevalence for fused teeth than European and European-derived population [7]. Due to this low prevalence, the importance of these anomalies tends to be underestimated. Prevalence of triple teeth (fusion of three teeth) is well documented in maxillary region [1, 3, 8] but its occurrence in mandibular anterior region of primary dentition is rare and uncommon.

 The exact aetiology is unknown, but it is thought to be due to impact of some physical forces or pressure on the developing tooth germs and subsequent union of enamel organ and the dental papilla resulting in fusion of teeth. Some authors believe it to be due to hereditary cause or excess administration of vitamin A, viral infection, or use of thalidomide drug during pregnancy [1, 3–6]. This fusion could be attributed to the decreased available space caused by the presence of supernumerary tooth and the proximity between tooth germs. This association suggests that there may be a single common attribute of the dental lamina predisposing to epithelial stripping and to laminal hyperactivity [9]. Recently Mitsiadis et al. [10] (2005) demonstrated that Notch signalling mediated through the *Jagged2* gene plays an essential role in tooth development and fusion of teeth.

 Often there is a difficulty in differentiating fusion and gemination. In case of fusion, one tooth is less than normal in the affected arch, but in case of gemination tooth number is unaffected. In fusion two teeth formed from two different teeth germs fuses during development and in gemination there is formation of two complete or incomplete teeth from single tooth germ. In gemination, crown is either totally or partially separated with single root or root canal [2, 4, 6]. Hence some authors considered the term, double teeth as most appropriate one for such condition [7]. Levitas classification can be practically helpful in distinguishing cases of fusion and gemination [11]. Radiographic examination is helpful in diagnosing and differentiating the fused teeth from gemination. 

 Fused teeth are usually asymptomatic but sometimes fused primary teeth can be associated with various clinical manifestations and anomalies of permanent dentition like conical or peg-shaped permanent tooth, ectopic eruption, delayed eruption, arch asymmetry, occlusal disturbance, tooth agenesis, hypodontia of permanent dentition, or presence of supernumerary tooth [1, 4, 6, 7]. Fused teeth often present with delayed exfoliation as seen in the present case [1]. Winter and Brook reported that the overall frequency of the permanent anomalies fallowing primary double tooth is 30–50% in Caucasian and 75% in Japanese [1]. Fused teeth are of aesthetic concern as they are wider than normal surrounding teeth and results into excess of dental space. Fused teeth commonly exhibit grooves at the site of union, which is difficult to clean and this can result in caries and periodontal problem [4].

 Management of fused teeth includes observation of normal exfoliation if asymptomatic, restoration of deep grooves, periodontal and orthodontic correction if required, endodontic therapy or extraction if symptomatic with pulpal involvement [7, 8]. Wu et al. (2010) suggested orthodontic treatment to fused and rotated teeth followed by complementary esthetic treatment [7]. 

## 4. Conclusion

 Due to lower prevalence of fused teeth, the importance of these anomalies tends to be underestimated. Fused teeth can result in various clinical manifestations on permanent dentition. Hence carful clinical and radiographic evaluation and monitoring is necessary.
